# Gestational Age and Socioeconomic Achievements in Young Adulthood: A Danish Population-Based Study

**DOI:** 10.1001/jamanetworkopen.2018.6085

**Published:** 2018-12-14

**Authors:** Josephine Funck Bilsteen, David Taylor-Robinson, Klaus Børch, Katrine Strandberg-Larsen, Anne-Marie Nybo Andersen

**Affiliations:** 1 Department of Pediatrics, Hvidovre University Hospital, Hvidovre, Denmark; 2 Section of Epidemiology, Department of Public Health, University of Copenhagen, Copenhagen, Denmark; 3 Department of Public Health and Policy, University of Liverpool, Liverpool, United Kingdom

## Abstract

**Importance:**

The poor health outcomes associated with preterm birth are well established. However, it is less clear how small variations in gestational age, even within the term range, are associated with long-term opportunities and well-being, as measured by socioeconomic outcomes in adulthood.

**Objective:**

To examine the association of gestational age at birth with educational achievement, income, and primary source of income in adulthood.

**Design, Setting, and Participants:**

This Danish population-based, register-based cohort study examined all live-born singletons born in Denmark from 1982 to 1986 without congenital anomalies and who lived in Denmark at age 28 years. Data analysis was conducted from November 2, 2017, to June 15, 2018.

**Exposures:**

Gestational age at birth in completed weeks (22-45 weeks).

**Main Outcomes and Measures:**

Educational attainment, personal income, and primary source of income at age 28 years.

**Results:**

In a population of 228 030 singletons (4.0% preterm, 12.1% early term; 49.4% female), 36.3% had a tertiary education at age 28 years. Among adults born at 22 to 27 weeks of gestation, 21.6% had a tertiary education, and 23.2% had an income in the highest tertile. Using 40 weeks of gestation as the reference, the adjusted odds ratio for tertiary education for individuals born at 22 to 27 weeks of gestation was 0.21 (95% CI, 0.13-0.35) and the corresponding figures for 28 to 31, 33, 36, 38, and 43 to 45 weeks of gestation were 0.45 (95% CI, 0.37-0.55), 0.67 (95% CI, 0.54-0.83), 0.84 (95% CI, 0.77-0.93), 0.85 (95% CI, 0.81-0.89), and 0.93 (95% CI, 0.83-1.04), respectively. The adjusted odds ratio for highest income tertile for individuals born at 22 to 27 weeks of gestation was 0.66 (95% CI, 0.41-1.06) and the corresponding figures for 28 to 31, 33, 36, 38, and 43 to 45 weeks of gestation were 0.80 (95% CI, 0.68-0.94), 0.77 (95% CI, 0.63-0.93), 0.89 (95% CI, 0.82-0.96), 0.95 (95% CI, 0.91-0.99), and 1.00 (95% CI, 0.91-1.12), respectively. All estimates were adjusted for sex, birth year, parity, maternal age, maternal education, and maternal country of origin.

**Conclusions and Relevance:**

Shorter gestational duration even within the term range was associated with poorer socioeconomic outcomes in adulthood. While adults born at 35 to 38 weeks of gestation experienced only slightly increased risk of adverse socioeconomic outcomes, this may have a significant impact on public health, since a large proportion of all children are born in these weeks.

## Key Points

**Question:**

How was the whole range of gestational age at birth associated with socioeconomic outcomes in adulthood (education, personal income, and primary source of income)?

**Findings:**

In this cohort study including 228 030 singletons, lower gestational age (<39 weeks of gestation) was associated with lower odds of high educational level and high personal income and increased odds of receiving disability pension and cash welfare benefits compared with individuals born at 40 weeks of gestation.

**Meaning:**

These findings suggest that lower gestational age even within the term range may have implications for long-term opportunities and well-being as measured by socioeconomic outcomes in adulthood.

## Teaser

This Danish population-based cohort study examines the associations between the entire span of gestational age at birth and socioeconomic outcomes, measured as educational achievements, personal income, and primary source of income, in young adulthood.

## Introduction

Worldwide, 1 in 10 children are born preterm (<37 weeks’ gestational age).^1^ Preterm birth is one of the leading causes of perinatal morbidity and mortality.^2^ Beyond the perinatal period, there is increasing recognition of the longer-term health and social sequelae of preterm birth, such as effects on independent living, quality of life, self-perception, and socioeconomic achievements.^3,4,5,6,7,8^

The association between the whole range of gestational age rather than preterm birth and social and health outcomes in adulthood are less thoroughly investigated. Children born from 37 to 41 weeks of gestation have traditionally been considered a low-risk and homogeneous group.^9^ However, emerging evidence suggests that the risk of adverse outcomes in childhood and adolescence varies even within the term range of gestational age.^10,11^ Recent studies have shown that children born early term (37-38 weeks) were more likely to have poorer school performance^10,11,12,13^ and cognitive outcomes^10,11^ compared with children born at 40 weeks of gestation or from 39 through 41 weeks of gestation. However, much less is known about the long-term socioeconomic achievements of adults born early term. The aim of this study was therefore to examine the associations between the entire span of gestational age and socioeconomic outcomes in the Danish population, measured as educational achievements, personal income, and primary source of income in young adulthood. In addition, the hypothesis that parental socioeconomic position might act as an effect modifier of the association between gestational age and socioeconomic outcomes was investigated.

## Methods

In this longitudinal register linkage cohort study of all singletons born in Denmark from 1982 through 1986, individuals were followed from birth to age 28 years in the Danish national registers. The study followed the Strengthening the Reporting of Observational Studies in Epidemiology (STROBE) reporting guideline. According to Danish legislation no ethical permission was required for register-based research; however, the study was approved by the local data protection authorities.

### Data Sources

All live-born singletons recorded in the Danish Medical Birth Register^14^ from 1982 through 1986 were included in this study. Individuals were included from 1982 as information on maternal education was available from 1981 (and maternal education 1 year before birth was included as a covariate). As we investigate socioeconomic outcomes at age 28 years, only individuals born in or before 1986 could be included as we had information on socioeconomic attainment up to 2014. Data from the Danish Medical Birth Registry^14^ were linked to data on education, income, and primary source of income from the Population Education Register,^15^ the Income Statistics Register,^16^ and the Employment Classification Module, respectively.^17^ Information on these socioeconomic variables was obtained in the calendar year a person turned 28 years old. Furthermore, information on the mothers’ education, age at birth, and country of origin was linked to the live-born singletons. The linkage across the different registers was enabled by the Danish system of unique person identifiers.

### Study Population

A total of 258 770 live-born singletons were recorded in the Danish Medical Birth Register within the study period. For the analyses, a total of 13 181 individuals (5.1%) with the following characteristics were excluded: individuals who died in or before the calendar year they turned 28 years old (n = 4100) and individuals with at least 1 congenital anomaly registered within the first year of life (n = 10 030). Congenital anomaly diagnoses were obtained from the Danish National Patient Register^18^ and congenital anomalies excluding minor congenital anomalies were defined according to the European Registration of Congenital Anomalies and Twins definitions.^19^ Furthermore, 11 804 individuals (4.8%) who did not live in Denmark throughout the calendar year they turned 28 years old were excluded (eTable 1 in the Supplement shows excluded individuals according to gestational age). Additionally, 18 individuals were excluded because their relationship between birth weight and gestational age were considered implausible according to the growth curves presented by Alexander and colleagues.^20^ In addition, a total of 5737 individuals (2.5%) who lacked information on at least 1 of the variables of interest were excluded (gestational age [n = 764], sex [n = 349], maternal country of origin [n = 10], maternal age [n = 5], maternal education [n = 3180], education [n = 1806], personal income [n = 660], primary source of income [n = 660]) (eTable 2 in the Supplement). Consequently the analysis population consisted of 228 030 live-born singletons (eFigure in the Supplement).

### Exposure, Outcomes, and Covariates

Information on the main exposure of interest, gestational age, was obtained from the Danish Medical Birth Register.^14^ The length of gestation was estimated by ultrasonography examination, last menstrual period, or clinical examination.^21^ Gestational age in completed weeks of gestation was categorized into the following groups: less than 22 to 28, 28 to 31, 32, 33, 34, 35, 36, 37, 38, 39, 40, 41, 42, and 43 to 45.

The main outcomes of interest were educational attainment, income, and source of income at age 28 years. The education variable was categorized according to the International Standard Classification of Education (ISCED) 2011 definitions^22^ in the following categories: primary (ISCED level: 1-2), secondary (ISCED level: 3-4), and tertiary (ISCED level: 5-8). Information on annual disposable personal income (total personal income excluding tax and interest expenses) in the calendar year a person turned age 28 years was categorized into tertiles for each calendar year. In the Employment Classification Module individuals were assigned a primary source of income based on their most important source of income throughout the year, ie, the activity with the highest incomes.^17^ Primary source of income at age 28 years was categorized into the following 5 groups: employed, unemployed, cash benefits, disability pension, and others*.* The category of employed included employed and self-employed persons, and the category of unemployed included persons who had been unemployed for 6 months or more in a given calendar year.^23^ The category of cash benefits included people who could not support themselves or their families and could not be supported by other benefits.^24^ The category of disability pension included people with a substantial and permanent reduced working capacity.^25^ The category of others included people whose primary source of income were not from employment, unemployment benefits, cash benefits, or disability pension, and included people whose primary sources of income were study grants and sickness leave benefits.

Other covariables of interest were birth year, sex, maternal age at birth, parity, maternal education 1 year before birth (categorized according to ISCED level), and maternal county of origin (Table 1). The maternal country of origin was defined as Denmark, other western country, or nonwestern country according to the classification by Statistics Denmark.^26^

**Table 1.** Perinatal and Sociodemographical Characteristics by Gestational Age in Completed Weeks in the Analysis Population

Characteristics	All Individuals, No. (%)	Gestational Age in Completed Weeks, %													
		<28	28-31	32	33	34	35	36	37	38	39	40	41	42	≥43
No.	228 030	125	915	542	679	1167	1921	3906	7890	19 688	39 138	89 484	41 571	18 345	2659
Sex															
Men	115 411 (50.6)	50.4	54.5	55.0	52.9	51.7	57.6	52.2	52.6	52.3	51.6	50.2	49.1	49.7	49.8
Women	112 619 (49.4)	49.6	45.5	45.0	47.1	48.3	42.4	47.8	47.4	47.7	48.4	49.8	50.9	50.3	50.2
Parity															
0	104 463 (45.8)	55.2	52.8	50.9	47.7	51.8	51.9	52.4	49.3	45.7	44.0	44.3	46.4	50.3	50.5
1	85 133 (37.3)	24.0	29.7	29.2	33.7	30.8	30.1	30.4	32.9	35.4	38.2	38.7	38.2	34.9	34.5
2	28 944 (12.7)	12.0	11.5	13.7	12.1	13.5	12.9	11.5	12.4	13.7	13.3	13.0	11.7	11.7	11.4
≥3	9490 (4.2)	8.8	6.0	6.3	6.5	3.9	5.0	5.6	5.4	5.1	4.5	4.0	3.6	3.1	3.6
Year of birth															
1982	45 456 (19.9)	20.8	20.0	18.3	16.6	18.5	17.3	19.3	19.6	18.5	18.0	21.2	20.2	20.2	17.6
1983	43 939 (19.3)	16.0	19.9	16.4	18.9	21.2	19.7	18.5	18.9	18.4	18.4	19.7	19.5	19.7	18.5
1984	44 555 (19.5)	27.2	16.5	22.7	22.1	19.3	21.3	20.1	20.5	20.2	20.6	19.7	18.5	17.6	17.3
1985	46 324 (20.3)	14.4	20.3	20.8	21.4	20.7	21.0	20.4	20.3	21.3	21.0	19.4	20.8	20.6	22.3
1986	47 756 (20.9)	21.6	23.3	21.8	21.1	20.3	20.7	21.7	20.7	21.7	22.0	20.0	21.0	22.0	24.3
Maternal age, y															
<20	8672 (3.8)	4.8	6.2	6.1	3.8	6.7	5.5	5.8	5.2	4.5	3.6	3.6	3.4	3.8	4.0
20-24	65 011 (28.5)	21.6	29.3	32.5	30.9	30.2	30.1	29.9	30.6	29.0	28.0	28.2	28.0	29.4	30.6
25-29	90 415 (39.7)	36.8	36.2	31.5	35.1	35.3	37.5	36.6	35.5	36.6	38.7	40.3	41.3	41.6	41.3
30-34	47 419 (20.8)	22.4	18.7	19.4	19.7	19.5	17.5	19.0	19.6	20.9	21.7	20.8	21.0	19.7	19.9
≥35	16 513 (7.2)	14.4	9.6	10.5	10.5	8.2	9.3	8.7	9.1	9.0	7.9	7.0	6.3	5.6	4.2
Maternal education															
Primary	105 196 (46.1)	50.4	54.3	56.1	53.0	54.3	53.5	52.7	51.9	49.2	46.5	45.4	43.8	44.5	49.5
Secondary	83 890 (36.8)	34.4	33.2	31.5	32.7	32.4	33.0	34.3	34.6	35.1	36.5	37.1	38.0	38.2	34.0
Tertiary	38 944 (17.1)	15.2	12.5	12.4	14.3	13.3	13.5	13.1	13.5	15.7	17.1	17.6	18.2	17.3	16.5
Maternal country of origin															
Denmark	218 831 (96.0)	NR^a^	95.8	94.8	95.1	95.8	95.2	95.1	94.9	94.8	95.4	96.0	96.8	97.0	96.7
Other western	3901 (1.7)	NR^a^	2.0	1.7	1.6	1.6	1.9	1.8	2.0	1.8	1.7	1.7	1.7	1.5	1.7
Nonwestern	5298 (2.3)	NR^a^	3.0	3.5	3.2	2.6	2.9	3.1	3.1	3.4	2.8	2.3	1.5	1.5	1.5

Abbreviation: NR, not reported.

^a^ Not reported since some cells counts were less than 5.

### Statistical Analysis

Multinomial logistic regression models were used to examine the associations between gestational age and education, income, and primary source of incomes at age 28 years in the analysis population. Adjusted odds ratio (aOR) and unadjusted OR from the multinomial logistic regression models were presented with 95% confidence intervals. Based on a priori confounder identification, the following covariables were adjusted for: birth year, sex, parity, maternal age, maternal country of origin, and maternal education. The study explored any potential interactions between gestational age and maternal educational level on the socioeconomic outcomes by including an interaction term in the multinomial logistic regressions. Statistical significance for interaction was assumed at an α level of .05. The *P* value for the interaction term between gestational age and maternal educational level did not reach statistical significance for any of the socioeconomic outcomes. The interaction terms were not included in the main analyses.

A sensitivity analysis was conducted in the oldest subsample of the population to test whether the associations differed if outcomes were measured at age 30 years. All statistical analyses were performed using SAS statistical software, version 9.4 (SAS Institute Inc) from November 2, 2017, to June 15, 2018.

## Results

In the analysis population of 228 030 individuals, 4.0% were born before 37 weeks of gestation, 12.1% were born from 37 through 38 weeks of gestation, 74.6% were born from 39 through 41 weeks of gestation, and 9.2% were born after 41 weeks of gestation. The sex distribution was 49.4% women and 50.6% men (Table 1).

Adults born before 39 weeks of gestation compared with adults born at 40 weeks of gestation had higher percentages of nulliparous mothers, mothers with parity higher than 3, mothers younger than 20 years and older than 34 years at participants’ birth, mothers with primary education, and mothers of nonwestern origin (Table 1).

Overall, 36.3% of the individuals had completed a tertiary eduation. Among individuals born at 40 weeks of gestation, 36.8% had completed a tertiary education; the corresponding figures for individuals born at 22 to 27, 28 to 31, 32, 33, 34, 35, 36, 37, 38, 39, 41, 42, and 43 to 45 weeks of gestation were 21.6%, 26.2%, 30.1%, 31.1%, 29.8%, 30.4%, 33.5%, 32.0%, 34.0%, 36.3%, 37.8%, 37.3%, and 34.9%, respectively (Table 2). The percentage of individuals in the highest income tertile was 33.6% among individuals born at 40 weeks of gestation and the corresponding figures for individuals born at 22 to 27, 28 to 31, 32, 33, 34, 35, 36, 37, 38, 39, 41, 42, and 43 to 45 weeks of gestation were 23.2%, 29.0%, 29.2%, 28.1%, 28.2%, 32.1%, 30.7%, 31.5%, 32.4%, 33.5%, 34.1%, 33.6%, and 33.1%, respectively (Table 2). The percentages of individuals with employment as their primary source of income was 71.6% among individuals born at 40 weeks of gestation and the corresponding figures for individuals born at 22 to 27, 28 to 31, 32, 33, 34, 35, 36, 37, 38, 39, 41, 42, and 43 to 45 weeks of gestation were 58.4%, 61.1%, 64.6%, 67.2%, 66.4%, 71.0%, 68.3%, 70.4%, 70.3% 71.6%, 71.8%, 71.4%, and 71.3%, respectively (Table 2). The percentages of individuals in the analysis population with disability pension, cash benefits, unemployment, and other as primary source of income were 1.9% (n = 4420), 5.6% (n = 12 660), 1.8% (n = 4063), and 19.4% (n = 44 268), respectively (Table 2).

**Table 2.** Socioeconomic Outcomes at Age 28 Years According to Gestational Age in the Analysis Population

Socioeconomic Outcomes	All Individuals, No. (%)	Gestational Age in Completed Weeks, %													
		<28	28-31	32	33	34	35	36	37	38	39	40	41	42	≥43
No.	228 030	125	915	542	679	1167	1921	3906	7890	19 688	39 688	91 320	42 338	18 671	2713
Education															
Primary	43 037 (18.9)	40.8	31.3	23.6	25.3	26.1	23.3	22.4	22.1	21.4	19.1	18.3	17.4	17.4	19.3
Secondary	102 284 (44.9)	37.6	42.5	46.3	43.6	44.0	46.3	44.0	45.9	44.5	44.6	44.9	44.8	45.3	45.8
Tertiary	82 709 (36.3)	21.6	26.2	30.1	31.1	29.8	30.4	33.5	32.0	34.0	36.3	36.8	37.8	37.3	34.9
Personal income															
Lowest tertile	76 008 (33.3)	34.4	36.5	35.2	37.0	37.4	33.7	34.8	34.4	34.3	33.3	33.2	32.5	33.3	32.9
Middle tertile	76 012 (33.3)	42.4	34.5	35.6	34.9	34.4	34.1	34.5	34.1	33.3	33.2	33.2	33.4	33.1	34.0
Highest tertile	76 010 (33.3)	23.2	29.0	29.2	28.1	28.2	32.1	30.7	31.5	32.4	33.5	33.6	34.1	33.6	33.1
Primary source of income															
Employment	162 619 (71.3)	58.4	61.1	64.6	67.2	66.4	71.0	68.3	70.4	70.3	71.6	71.6	71.8	71.4	71.3
Unemployment	4063 (1.8)	0.0	1.2	1.3	1.9	1.9	1.9	2.2	1.9	1.9	1.9	1.8	1.6	1.8	2.5
Cash benefits	12 660 (5.6)	7.2	8.6	7.9	8.2	8.2	6.4	6.8	6.7	6.4	5.5	5.4	5.0	5.2	5.6
Disability pension	4420 (1.9)	17.6	11.9	6.6	5.7	4.3	3.1	2.9	2.2	2.2	1.9	1.7	1.7	1.8	2.4
Other^a^	44 268 (19.4)	16.8	17.2	19.6	16.9	19.2	17.6	19.8	18.9	19.1	19.1	19.5	19.9	19.8	18.2

^a^ Other includes study grants and sickness leave.

**Figure 1.**
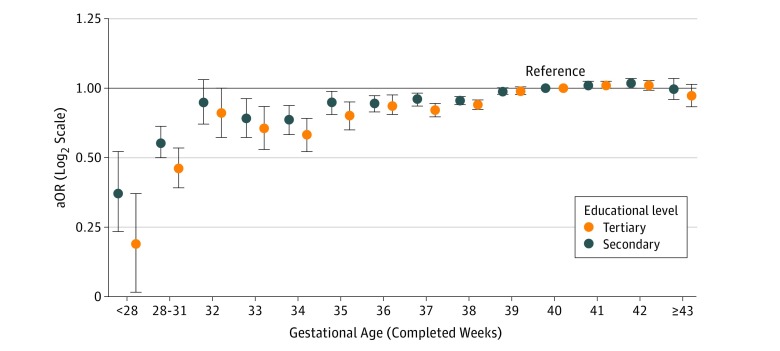
Adjusted Odds Ratios (aORs) With 95% CIs for Tertiary and Secondary Education by Gestational Age The aORs were adjusted for sex, birth year, parity, maternal education, and maternal country of origin.

Figure 1 shows that the aOR of having completed tertiary and secondary education at the age of 28 years increased with increasing gestational age at birth for individuals born before 40 weeks of gestation (eTable 3 in the Supplement). The aOR for tertiary education for individuals born before 28 weeks of gestation was 0.21 (95% CI, 0.13-0.35) and the corresponding figures for 28 to 31, 32, 33, 34, 35, 36, 37, 38, 39, 41, 42, and 43 to 45 weeks of gestation were 0.45 (95% CI, 0.37-0.55), 0.78 (95% CI, 0.61-1.00), 0.67 (95% CI, 0.54-0.83), 0.63 (95% CI, 0.53-0.74), 0.76 (95% CI, 0.66-0.87), 0.84 (95% CI, 0.77-0.93), 0.80 (95% CI, 0.75-0.86), 0.85 (95% CI, 0.81-0.89), 0.97 (95% CI, 0.94-1.01), 1.03 (95% CI, 1.00-1.07), 1.03 (95% CI, 0.98-1.08), and 0.93 (95% CI, 0.83-1.04), respectively. For secondary education, the aORs were slightly attenuated compared with the aORs for tertiary education.

**Figure 2.**
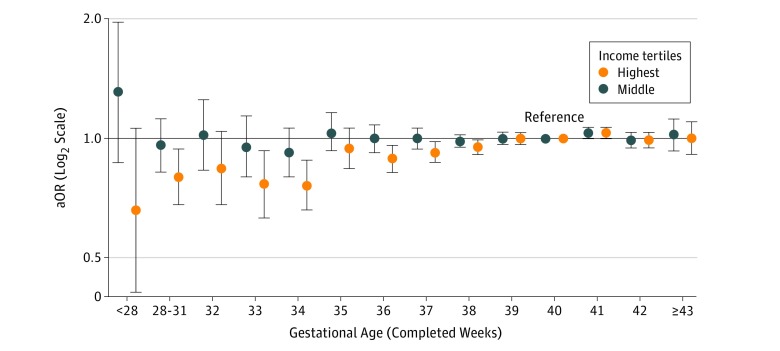
Adjusted Odds Ratios (aORs) With 95% CIs for Highest and Middle Income Tertiles by Gestational Age The aORs were adjusted for sex, birth year, parity, maternal education, and maternal country of origin.

Figure 2 shows that young adults born before 39 weeks of gestation were less likely to have a personal income in the highest income tertile compared with adults born at 40 weeks of gestation (eTable 4 in the Supplement). The aOR for highest income tertile for individuals born before 28 weeks of gestation was 0.66 (95% CI, 0.41-1.06) the corresponding figures for 28 to 31, 32, 33, 34, 35, 36, 37, 38, 39, 41, 42, and 43 to 45 weeks of gestation were 0.80 (95% CI, 0.68-0.94), 0.84 (95% CI, 0.68-1.04), 0.77 (95% CI, 0.63-0.93), 0.76 (95% CI, 0.66-0.88), 0.94 (95% CI, 0.84-1.06), 0.89 (95% CI, 0.82-0.96), 0.92 (95% CI, 0.87-0.98), 0.95 (95% CI, 0.91-0.99), 1.00 (95% CI, 0.97-1.03), 1.03 (95% CI, 1.00-1.06), 0.99 (95% CI, 0.95-1.03), and 1.00 (95% CI, 0.91-1.12), respectively. No association between gestational age and having a personal income in the middle income tertile was observed (Figure 2; eTable 4 in the Supplement).

**Figure 3.**
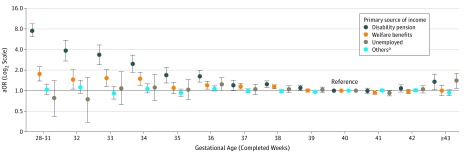
Adjusted Odds Ratios (aORs) With 95% CIs for Primary Source of Income Categories by Gestational Age The aORs were adjusted for sex, birth year, parity, maternal education, and maternal country of origin. ^a^The category “others” included people whose primary source of income were not from employment, unemployment benefits, cash benefits, or disability pension.

Figure 3 shows that adults born before 39 weeks of gestation were more likely to have cash benefits and disability pension as a primary source of income compared with adults born at 40 weeks of gestation (eTable 5 in the Supplement). In addition, adults born after 42 weeks of gestation had an increased aOR of disability pension, and this finding was statistically significant. No clear associations were observed between gestational age and unemployment and the group “other.” However, adults born after 42 weeks of gestation had a higher aOR of being unemployed.

Overall adjustment for potential confounders attenuated the associations (eTable 3-5 in the Supplement). When one of the selected confounders was omitted from the models, the estimates only changed slightly with the exception of maternal education, which attenuated the estimates for educational level, disability pension, and welfare benefits to a greater extent than the other selected confounders. There were no notable differences between the findings from the main analysis and the findings when the socioeconomic outcomes were measured at age 30 years in the oldest subsample of the analysis population.

## Discussion

In this total population study of singletons born in Denmark, preterm birth and early term birth were associated with poorer socioeconomic outcomes in young adulthood. Lower gestational age (before 39 weeks of gestation) was associated with increased odds of disability pension and cash benefits, and decreased odds of having a secondary and tertiary education and being in the highest income tertile. Adults born at 41 weeks of gestation had similar or slightly better socioeconomic outcomes compared with adults born at 40 weeks of gestation. Adults born postterm did not differ from those born at 40 weeks of gestation in terms of income and educational level. However, increased odds of disability pension and unemployment were observed for adults born after 42 weeks of gestation.

### Comparison With Other Studies

To our knowledge, this is the first study to show associations with early term birth and worse socioeconomic outcomes in adulthood. A previous Swedish study that investigated early term birth did not observe significantly poorer socioeconomic outcomes in adulthood among these adults compared with adults born from 39 to 41 weeks of gestation.^6^ Adults with at least 1 indication of disability were excluded from most of the analyses in the Swedish study, and this exclusion might explain the different findings since a strong gestational age gradient in individuals with an indication of disability was observed. In the present study we did not exclude individuals with indications of disabilities since disability may be a consequence of lower gestational age.^2^

Our findings regarding the socioeconomic outcomes of adults who had been born preterm corroborate a recent meta-analysis^27^ that found that preterm birth and/or low birth weight was associated with lower educational qualifications and an increased likelihood of welfare benefits. Previous studies also found that adults who had been born preterm were less likely to have higher incomes^4,6 ^and more likely to receive disability pension.^7,8^ We did not find an association between preterm birth and unemployment in our study. This finding was consistent with previous Nordic studies.^6,7,8^ These findings were perhaps surprising since the unemployment rate often differs by educational level^28^ and gestational age differences were observed for educational level, but it could be explained by selection of the most health challenged and/or socially challenged preterm offspring into other welfare benefits.

We did not find that preterm birth had differential socioeconomic consequences in adulthood on the basis of maternal educational level. A previous Swedish study^29^ suggested that the association of preterm birth with school performance was more severe in those with lower parental socioeconomic position. By contrast, our test for interaction between gestational age and maternal educational level on the 3 socioeconomic outcomes did not suggest that maternal education was an effect modifier. However, it cannot be ruled out that effect modification by this or other measures of parental socioeconomic position would be present in some strata.

### Strengths and Limitations

The study was based on routinely collected population-covering data and examined socioeconomic achievements across the whole gestational age spectrum. The Danish registers used in this study were nationwide and had a high level of completeness.^14,15,16,17^ The obstetric and socioeconomic data were obtained from the routine population-based data, which obviates recall bias and limits selection bias. Linkage across several Danish registers at an individual level made it possible to follow individuals from birth into young adulthood. The use of several indicators for socioeconomic position in young adulthood was an additional strength of this study.

The accuracy of gestational age estimates is likely to be influenced by distinct methods of estimating gestational age^30^ and registration of gestational age.^21^ However, the prospective design reassures that this misclassification is nondifferential. Individuals with implausible relationships of gestational age and birth weight were excluded to reduce the problem of registration errors. A study reported that the majority of children in the Medical Birth Register had reported 1 additional week of gestation compared with the gestational age reported in the medical records in a sample from 1982 to 1988.^21^ The distribution of discrepancies in gestational age was the same across the whole range of gestational age and thereby this potential misclassification may have shifted the findings of this study to higher gestational ages, but would not have altered the observed associations.

Maternal educational level 1 year before delivery was used as an indicator of socioeconomic position. However, maternal education is not likely to capture all aspects of parental socioeconomic position,^31^ and as a consequence we may not have fully accounted for parental socioeconomic position. However, in a previous study we found maternal education to be the strongest socioeconomic indicator for preterm birth.^32^ A potential limitation was the changing gestational age distribution over time, whereby the proportion of children born before 37 weeks of gestation was higher in the Danish population of live-born children born from 2012 to 2016 compared with our study population (eTable 6 in the Supplement). In addition, the survival and treatment of children born preterm has changed substantially from the study period until today.^1^ This was particularly important in regard to children born before 28 weeks of gestation for whom the survival rate had increased considerably over the last decades. As a consequence, generalizing the findings from this study to later cohorts of very and extremely preterm children should be made with caution. The findings related to late preterm and early term children, namely a graded association, where even 2 weeks might matter, was probably affected to a lesser extent by this limitation than children born very preterm.

Future studies should focus on identifying mechanisms underlying the association between gestational age and socioeconomic outcomes. Although beyond the scope of our study, some of the plausible mechanisms may be maturation outside the uterine milieu, the morbidity associated with lower gestational age, and the underlying causes of premature birth.^33^ Children born at different weeks of gestation may have distinct maturational trajectories of the brain as brain growth rapidly increases in the last trimester.^34^ A 5-fold increase in white matter volume occurs between 35 and 41 weeks of gestation.^34^ Studies indicate that the morphology of the brain in childhood differs with gestational age^35^ even within the term range of gestational age.^36^ The increasing morbidity with decreasing gestational age may also influence socioeconomic position. In addition, it cannot be ruled out that factors causing parturition might also play a role in the neurodevelopment and through this process possibly also socioeconomic outcomes in adulthood. The primary causes of labor in the majority of births are unknown.^2^ However, infants born preterm have higher rates of congenital malformations, intrauterine growth restriction, chorioamnionitis, maternal smoking, and high-risk pregnancies (preeclampsia, hypertension, and diabetes).^33^ Each of these factors could potentially be associated with poor neurodevelopmental outcomes.^33^

Given that so many powerful factors affect socioeconomic position from birth to adulthood, the findings of poorer socioeconomic outcomes of adults born as little as 2 weeks before term (40 weeks of gestation) are noteworthy. The study’s findings suggest that by addressing gestational age as a continuum, one may provide insight into the association between gestational age and long-term outcomes, which could not have been obtained by investigating the differences between children born at the earliest gestational ages and children born at term. If these findings were causal, they would be of relevance to clinicians and for future research since a large proportion of children are born in the weeks before term (40 weeks of gestation). To address causality, randomized trials with long-term follow-up of labor induction vs postponement of labor induction are needed. In addition, the study’s findings emphasize the importance of early life factors for later life and further emphasize the need for tackling the socioeconomic gradients in preterm birth.

## Conclusions

Our study shows that not only adults born before 37 weeks of gestation but also adults born at 37 and 38 weeks of gestation have increased risks of several poorer socioeconomic outcomes including educational level, personal income, and primary source of income at age 28 years.

## Notes

Supplement. **eFigure.** Flow Charge of the Study Population **eTable 1.** Mortality, Congenital Anomalies and Emigration According to Gestational Age **eTable 2.** Characteristics in Study Population (including missing) and in Analysis Population **eTable 3.** Odds Ratios (OR) of Secondary and Tertiary Education by Gestational Age **eTable 4.** Odds Ratios (OR) of Middle and Highest Income Tertiles by Gestational Age **eTable 5.** Odds Ratios (OR) of Primary Source of Income Categories by Gestational Age **eTable 6.** Gestational Age Distribution in the Population Born From 2012-2016 and in Our Study Population
