# Supplementary material for: Gestational Age and Socioeconomic Achievements in Young Adulthood: A Danish Population-Based Study
Source: JAMA Netw Open. 2018 Dec 14;1(8):e186085. doi: 10.1001/jamanetworkopen.2018.6085 (PMC6324359; doi:10.1001/jamanetworkopen.2018.6085)
Supplement: Supplement. — eFigure. Flow Charge of the Study Population eTable 1. Mortality, Congenital Anomalies and Emigration According to Gestational Age eTable 2. Characteristics in Study Population (including missing) and in Analysis Population eTable 3. Odds Ratios (OR) of Secondary and Tertiary Education by Gestational Age eTable 4. Odds Ratios (OR) of Middle and Highest Income Tertiles by Gestational Age eTable 5. Odds Ratios (OR) of Primary Source of Income Categories by Gestational Age eTable 6. Gestational Age Distribution in the Population Born From 2012-2016 and in Our Study Population [file jamanetwopen-1-e186085-s001.pdf]

Supplementary Online Content

Bilsteen JF, Taylor-Robinson D, Børch K, Strandberg-Larsen K, Nybo Andersen A-M. Gestational age and socioeconomic achievements in young adulthood: a Danish population-based study. *JAMA Netw Open*. 2018;1(8):e186085. doi:10.1001/jamanetworkopen.2018.6085

eFigure. Flow Charge of the Study Population
eTable 1. Mortality, Congenital Anomalies and Emigration According to Gestational Age
eTable 2. Characteristics in Study Population (including missing) and in Analysis Population
eTable 3. Odds Ratios (OR) of Secondary and Tertiary Education by Gestational Age
eTable 4. Odds Ratios (OR) of Middle and Highest Income Tertiles by Gestational Age
eTable 5. Odds Ratios (OR) of Primary Source of Income Categories by Gestational Age
eTable 6. Gestational Age Distribution in the Population Born From 2012-2016 and in Our Study Population

This supplementary material has been provided by the authors to give readers additional information about their work.

© 2018 Bilsteen JF et al. *JAMA Network Open*.

eFigure. Flow Charge of the Study Population

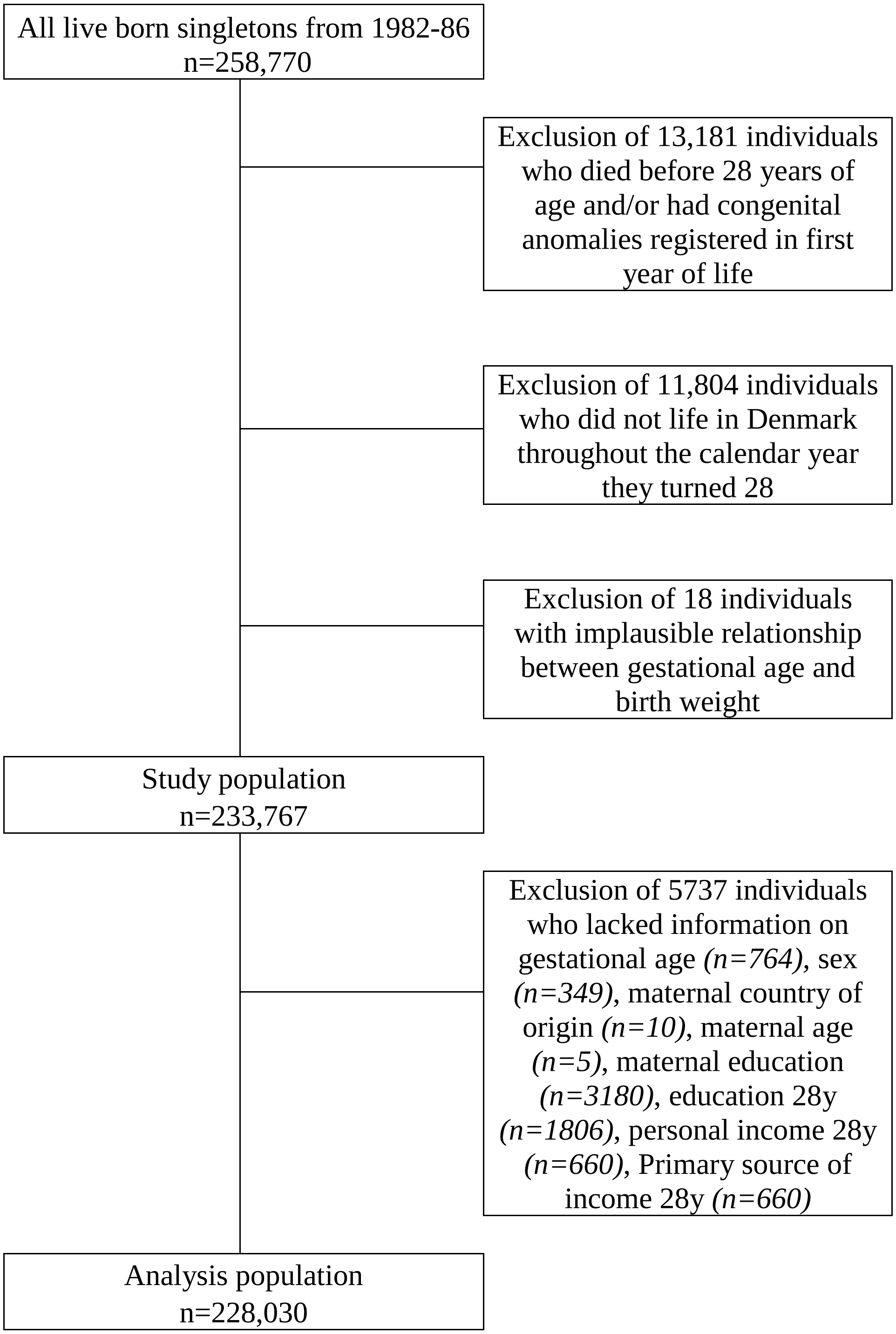

© 2018 Bilsteen JF et al. *JAMA Network Open*.

eTable 1. Mortality, Congenital Anomalies and Emigration According to Gestational Age

Gestational age (weeks)	All live born singletons 1982-86	Mortality from 0-28 y[a]		Congenital anomalies		Lived abroad at 28 y[b]	
	n	n	%	n	%	n	%
<28	426	260	61.0	34	8.0	8	4.8
28-32	1368	282	20.6	143	10.5	47	4.3
32	716	73	10.2	74	10.3	25	3.9
33	864	68	7.9	85	9.8	39	4.9
34	1441	74	5.1	134	9.3	65	4.8
35	2296	89	3.9	166	7.2	106	4.8
36	4638	142	3.1	335	7.2	225	5.0
37	9131	193	2.1	500	5.5	383	4.3
38	22,545	354	1.6	996	4.4	1114	5.2
39	44,219	543	1.2	1641	3.7	2174	5.0
40	100,204	1191	1.2	3412	3.4	4640	4.7
41	46,563	544	1.2	1582	3.4	2276	5.0
42	20,514	235	1.1	758	3.7	932	4.6
>42	2958	23	0.8	119	4.0	114	3.9
missing	887	29	3.3	51	5.7	52	6.1
	258,770	4100	1.6	10,030	3.9	12,200	4.8

[a]Mortality from birth up to and including the calendar year a person turned 28 years
[b]Individuals who lived abroad in or in part of the calendar year they turned 28 years. Percentages are based on a population of all individuals who were alive throughout the calendar year they turned 28 years.

© 2018 Bilsteen JF et al. *JAMA Network Open*.

eTable 2. Characteristics in study population (including missing) and in analysis population

		Study population		Analysis population	
		N=233,767		N=228,030	
		n	%	n	%
Gestational age (weeks)					
	<28	136	0.1	125	0.1
	28-32	936	0.4	915	0.4
	32	571	0.2	542	0.2
	33	699	0.3	679	0.3
	34	1207	0.5	1167	0.5
	35	1982	0.9	1921	0.8
	36	4022	1.7	3906	1.7
	37	8142	3.5	7890	3.4
	38	20,221	8.7	19,688	8.6
	39	40,045	17.1	39,138	17.1
	40	91,320	39.1	89,484	39.1
	41	42,338	18.1	41,571	18.2
	42	18,671	8.0	18,345	8.0
	>42	2713	1.2	2659	1.2
	missing	764	0.3	-	-
Sex					
	Female	114,939	49.2	112,619	49.4
	Male	118,479	50.7	115,411	50.6
	missing	349	0.2	-	-
Parity					
	0	106,639	45.6	104,463	45.8
	1	87,156	37.3	85,133	37.3
	2	29,914	12.8	28,944	12.7
	≥3	10,058	4.3	9490	4.2
	missing	-	-	-	-
Year of birth					
	1982	46,551	19.9	45,456	19.9
	1983	45,050	19.3	43,939	19.3
	1984	45,685	19.5	44,555	19.5
	1985	47,510	20.3	46,324	20.3
	1986	48,971	21	47,756	21
	missing	-	-	-	-
Maternal age					
	<20	9183	3.9	8670	3.8
	20-24	66,785	28.6	65,011	28.5
	25-29	92,481	39.6	90,415	39.7
	30-34	48,283	20.7	47,419	20.8

© 2018 Bilsteen JF et al. *JAMA Network Open.*

	≥35	17,030	7.3	16,513	7.2
	missing	5	<0.1	-	-
Maternal education					
	Primary	106,825	45.7	105,196	46.1
	Secondary	84,551	36.2	83,890	36.8
	Tertiary	39,211	16.8	38,944	17.1
	missing	3180	1.4	-	-
Maternal country of origin					
	Denmark	223,107	95.4	218831	96
	Other Western	4227	1.8	3901	1.7
	Non-Western	6423	2.8	5298	2.3
	missing	10	<0.1	-	-
Eduaction					
	Primary	44,477	19	43,037	18.9
	Secondary	103,920	44.5	102,284	44.9
	Tertiary	83,564	35.8	82,709	36.3
	missing	1806	0.8	-	-
Primary source of income					
	Empoyment	165,293	70.7	162,619	71.3
	Unemployment	4241	1.8	4,063	1.8
	Cash benefits	13,440	5.8	12,660	5.6
	Disability pension	4904	2.1	4,420	1.9
	Other[a]	45,229	19.4	44,268	19.4
	missing	660	0.3	-	-
Personal income[c]		Median: 187,390	Q25;Q75[b]: 134,541;230,215	Median: 188,240	Q25;Q75[b]: 135,693;230,758

[a]Other includes study grants sickness leave, maternity and paternity leave.
[b]Q25 is the lower quartile and Q75 is the higher quartile
[c]Median and quartiles are reported for personal annual income in Danish Kroner. The number of individuals with missing information in the study population was 660 (0.3%)

© 2018 Bilsteen JF et al. *JAMA Network Open.*

eTable 3. Odds Ratios (OR) of Secondary and Tertiary Education by Gestational Age

	Secondary education					Tertiary education				
	**OR**	**CI 95 %**		**OR[a]**	**CI 95 %**	**OR**	**CI 95 %**		**OR[a]**	**CI 95%**
GA (weeks)										
22-27	0.38	0.25-0.56		0.35	0.24-0.53	0.26	0.17-0.42		0.21	0.13-0.35
28-31	0.56	0.48-0.65		0.58	0.50-0.68	0.42	0.35-0.50		0.45	0.37-0.55
32	0.80	0.65-0.99		0.87	0.70-1.09	0.63	0.50-0.80		0.78	0.61-1.00
33	0.70	0.58-0.85		0.74	0.61-0.90	0.61	0.50-0.75		0.67	0.54-0.83
34	0.69	0.60-0.79		0.73	0.63-0.84	0.57	0.49.0.66		0.63	0.53-0.74
35	0.81	0.72-0.91		0.87	0.77-0.97	0.65	0.57-0.74		0.76	0.66-0.87
36	0.80	0.74-0.87		0.86	0.79-0.93	0.75	0.68-0.81		0.84	0.77-0.93
37	0.85	0.80-0.90		0.90	0.84-0.95	0.72	0.68-0.77		0.80	0.75-0.86
38	0.85	0.81-0.88		0.89	0.85-0.92	0.79	0.76-0.83		0.85	0.81-0.89
39	0.95	0.92-0.99		0.97	0.94-1.00	0.95	0.91-0.98		0.97	0.94-1.01
40	1.00	[Reference]		1.00	[Reference]	1.00	[Reference]		1.00	[Reference]
41	1.05	1.02-1.09		1.03	1.00-1.07	1.08	1.05-1.12		1.03	1.00-1.07
42	1.06	1.02-1.11		1.05	1.00-1.10	1.07	1.02-1.12		1.03	0.98-1.08
≥ 43	0.97	0.87-1.07		0.99	0.89-1.10	0.90	0.81-1.00		0.93	0.83-1.04

[a]Adjusted for sex, birth year, parity, maternal age, maternal education, and maternal country of origin

© 2018 Bilsteen JF et al. *JAMA Network Open*.

eTable 4. Odds Ratios (OR) of Middle and Highest Income Tertiles by Gestational Age

		Middle income tertile					Highest income tertile				
		**OR**	**CI 95%**		**OR[a]**	**CI 95 %**	**OR**	**CI 95%**		**OR[a]**	**CI 95%**
GA (weeks)											
22-27		1.26	0.84-1.88		1.31	0.87-1.96	0.65	0.40-1.04		0.66	0.41-1.06
28-31		0.95	0.81-1.11		0.96	0.82-1.12	0.79	0.67-0.92		0.80	0.68-0.94
32		1.01	0.83-1.24		1.02	0.83-1.25	0.82	0.66-1.01		0.84	0.68-1.04
33		0.95	0.79-1.13		0.95	0.80-1.14	0.75	0.62-0.91		0.77	0.63-0.93
34		0.93	0.81-1.06		0.92	0.80-1.06	0.75	0.65-0.86		0.76	0.66-0.88
35		1.02	0.91-1.13		1.03	0.93-1.16	0.94	0.84-1.05		0.94	0.84-1.06
36		0.99	0.92-1.07		1.00	0.92-1.08	0.88	0.81-0.95		0.89	0.82-0.96
37		0.99	0.94-1.05		1.00	0.94-1.06	0.91	0.86-0.96		0.92	0.87-0.98
38		0.97	0.94-1.01		0.98	0.95-1.02	0.94	0.90-0.97		0.95	0.91-0.99
39		1.00	0.97-1.03		1.00	0.97-1.03	1.00	0.97-1.02		1.00	0.97-1.03
40		1.00	[Reference]		1.00	[Reference]	1.00	[Reference]		1.00	[Reference]
41		1.03	1.00-1.06		1.03	1.00-1.06	1.04	1.01-1.07		1.03	1.00-1.06
42		1.00	0.96-1.04		0.99	0.95-1.03	1.00	0.96-1.04		0.99	0.95-1.03
≥ 43		1.04	0.94-1.14		1.02	0.93-1.12	1.00	0.90-1.09		1.00	0.91-1.12

[a]Adjusted for sex, birth year, parity, maternal age, maternal education, and maternal country of origin

© 2018 Bilsteen JF et al. *JAMA Network Open.*

eTable 5. Odds Ratios (OR) of Primary Source of Income Categories by Gestational Age

	**Disability pension**					**Cash benefits**				
	**OR**	**CI 95 %**		**OR[a]**	**CI 95 %**	**OR**	**CI 95 %**		**OR[a]**	**CI 95 %**
GA (weeks)										
28-31	8.14	6.59-10.05		7.62	6.16-9.44	1.87	1.47-2.37		1.76	1.39-2.24
32	4.29	3.03-6.06		3.86	2.72-5.47	1.63	1.18-2.23		1.46	1.06-2.02
33	3.56	2.56-4.96		3.34	2.40-4.66	1.62	1.23-2.15		1.52	1.15-2.02
34	2.69	2.01-3.60		2.48	1.85-3.32	1.64	1.32-2.03		1.49	1.20-1.86
35	1.83	1.41-2.39		1.69	1.30-2.20	1.19	0.99-1.44		1.10	0.91-1.33
36	1.75	1.44-2.13		1.62	1.34-1.98	1.32	1.16-1.50		1.21	1.06-1.38
37	1.28	1.09-1.51		1.20	1.02-1.41	1.26	1.15-1.38		1.16	1.06-1.28
38	1.30	1.17-1.45		1.25	1.12-1.39	1.20	1.13-1.29		1.14	1.07-1.22
39	1.12	1.02-1.22		1.10	1.01-1.21	1.02	0.97-1.08		1.01	0.96-1.06
40	1.00	[Reference]		1.00	[Reference]	1.00	[Reference]		1.00	[Reference]
41	0.98	0.89-1.07		1.00	0.91-1.09	0.92	0.87-0.97		0.94	0.89-0.99
42	1.07	0.95-1.20		1.09	0.96-1.22	0.96	0.90-1.04		0.98	0.91-1.05
≥ 43	1.38	1.07-1.79		1.35	1.04-1.75	1.05	0.88-1.24		1.01	0.85-1.20
	**Unemployment**					**Other primary sources of income[b]**				
	**OR**	**CI 95 %**		**OR[a]**	**CI 95%**	**OR**	**CI 95 %**		**OR[a]**	**CI 95%**
GA (weeks)										
28-31	0.81	0.44-1.47		0.77	0.42-1.40	1.03	0.86-1.23		1.05	0.88-1.25
32	0.82	0.39-1.73		0.74	0.35-1.57	1.11	0.90-1.38		1.14	0.92-1.42
33	1.17	0.67-2.03		1.09	0.63-1.90	0.93	0.76-1.14		0.93	0.76-1.15
34	1.16	0.76-1.78		1.12	0.73-1.72	1.06	0.92-1.23		1.08	0.93-1.26
35	1.11	0.80-1.55		1.04	0.75-1.45	0.91	0.81-1.03		0.94	0.83-1.06
36	1.32	1.06-1.64		1.24	1.00-1.55	1.07	0.98-1.16		1.08	1.00-1.18
37	1.11	0.93-1.31		1.05	0.88-1.24	0.99	0.93-1.05		1.00	0.94-1.06
38	1.12	1.00-1.25		1.05	0.94-1.18	1.00	0.96-1.04		1.00	0.96-1.04

© 2018 Bilsteen JF et al. *JAMA Network Open*.

39	1.08	0.99-1.18		1.04	0.95-1.13	0.98	0.95-1.01		0.97	0.94-1.00
40	1.00	[Reference]		1.00	[Reference]	1.00	[Reference]		1.00	[Reference]
41	0.90	0.82-0.99		0.92	0.84-1.01	1.02	0.99-1.05		1.01	0.98-1.04
42	1.02	0.91-1.16		1.05	0.93-1.18	1.02	0.98.1-06		1.02	0.98-1.06
≥ 43	1.42	1.11-1.83		1.40	1.09-1.80	0.94	0.85-1.04		0.94	0.85-1.04

[a]Adjusted for sex, birth year, parity, maternal age, maternal education, and maternal country of origin

[b]Other includes study grants and sickness leave.

© 2018 Bilsteen JF et al. *JAMA Network Open*.

eTable 6. Gestational Age Distribution in the Population Born From 2012-2016 and in Our Study Population

	Live born children 1982-86			Live born children 2012-16[a]		
**Gestational age**	**n**	**%**		**n**	**%**	
<28	426	0.16		1093	0.37	
28-31	1368	0.53		1881	0.64	
32-36	9955	3.85		15433	5.24	
37-40	176099	68.05		199681	67.81	
41+	70035	27.06		72832	24.73	
Missing	887	0.34		3569	1.21	
Total	258770			294489		

[a]The gestational age distribution from 2012-16 was obtained from Sundhedsdatastyrelsen. Fødsler og fødte Avanceret. http://www.esundhed.dk/sundhedsregistre/MFR/Sider/MFR06A.aspx. Accessed October 3, 2018

© 2018 Bilsteen JF et al. *JAMA Network Open*.
